# Correction: Seroprevalence of SARS-CoV-2 antibodies and retrospective mortality in a refugee camp, Dagahaley, Kenya

**DOI:** 10.1371/journal.pone.0299117

**Published:** 2024-02-14

**Authors:** Etienne Gignoux, Frida Athanassiadis, Ahmed Garat Yarrow, Abdullahi Jimale, Nicole Mubuto, Carole Déglise, Denis Onsongo Mosoti, Andrew S. Azman, Matilu Mwau, Francisco Luquero, Iza Ciglenecki

There are errors in the Funding and Competing Interests statements. The correct statements are:

**Funding:** The study was funded by the Operational Centre of Geneva of Médecins Sans Frontières (MSF). MSF programmatic funding covered all other costs associated with the study, including transportation, accommodations, materials, and equipment. MSF provided support in the form of salaries for FA, AGY, NM, CD, AA, and IC, and indirectly provided salary support for Epicentre employees EG and FL. The funder was involved in study design, data collection and analysis, decision to publish and preparation of the manuscript. This is indicated by the affiliation of the coauthors, and their specific roles are articulated in the ‘author contributions’ section.

**Competing Interests:** The authors have read the journal’s policy and have the following competing interests to declare: MSF provided support in the form of salaries for FA, AGY, NM, CD, AA, and IC, and indirectly provided salary support for Epicentre employees EG and FL. This does not alter our adherence to *PLOS ONE* policies on sharing data and materials. There are no patents, products in development, or marketed products associated with this research to declare.
