# Supplementary material for: Seroprevalence of SARS-CoV-2 antibodies and retrospective mortality in a refugee camp, Dagahaley, Kenya
Source: PLoS One. 2021 Dec 17;16(12):e0260989. doi: 10.1371/journal.pone.0260989 (PMC8683031; doi:10.1371/journal.pone.0260989)
Supplement: Attachment — Submitted filename: Comments.pdf [file pone.0260989.s003.pdf]

Summary:

The authors of this paper investigated the seroprevalence of SARS-CoV-2 antibodies and estimated the mortality in a refugee camp located in Dadaab refugee complex, Kenya. Rapid immunologic assay (BIOSYNEX® COVID-19 BSS [IgG/IgM] were used to determine seroprevalence. The authors interviewed all households (n=12860) in the camp to report the mortality rate. The study identified age groups that were most affected and observed an increase in the mortality rate during the pandemic.

Major comments:

1. The authors said “During the survey, CHWs recorded the size of all households (n=12,860) living in Dagahaley and estimated the population of Dagahaley at 88,793 inhabitants.” They also reports “ In total, we included 1206 participants in the seroprevalence study”. So, this means they have sampled 1.35 % of the entire population, therefore this study is not broad enough. To make more informed decision the authors should increase the sampling %.

2. It is quite surprising that the seroprevalence found this survey is significantly lower than Nairobi population which reported a seroprevalence of 34.7%. I am wondering if the authors have cross verified the rapid antibody test negative subjects with an ELISA test.

Minor comments:

1. Line 48: change where to were.
